# Villous Adenoma of the Appendix with Dysplasia

**DOI:** 10.4103/1319-3767.37807

**Published:** 2008-01

**Authors:** Pragati Karmarkar, Archana Joshi, Anne Wilkinson, Sadhana Mahore, Kalpana Bothale

**Affiliations:** Department of Pathology, NKP Salve Institute of Medical Sciences and Research Centre, Hingna, Nagpur - 440 019, Maharashtra, India. E-mail: rajuwilk_ngp@sancharnet.in

Sir,

A 35-year-old male patient reported to the surgeon with complaints of repeated attacks of pain in abdomen, associated with nausea and vomiting. He had clinical evidence of acute appendicitis. The patient was operated and an appendicectomy was done. The specimen was sent for histopathological examination.

Grossly, the appendix measured 6 × 1.2 × 1 cm and showed a bulbous, globular and enlarged tip. The cut section showed localized thickened wall at the tip.

**Figure 1:**
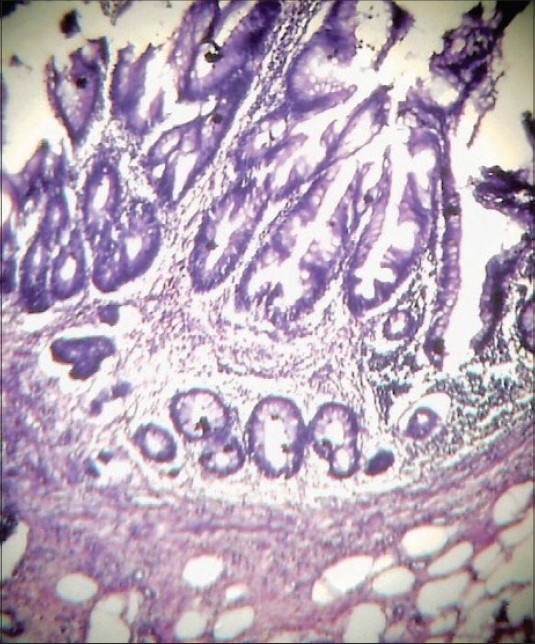
Photomicrograph showing part of wall of appendix with inflammation and villous configuration of the mucosa (Haematoxylin and eosin, ×10)

**Figure 2:**
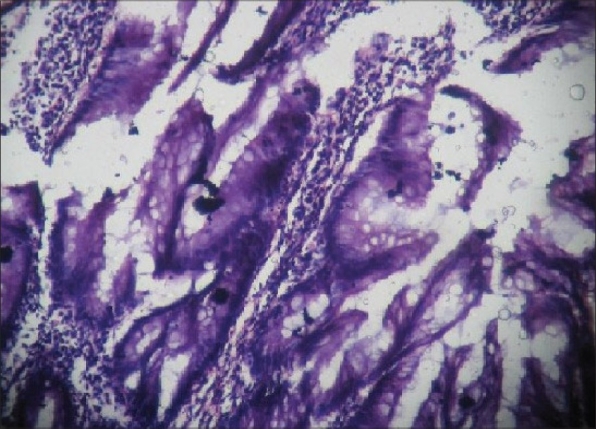
Photomicrograph of the epithelium of appendix showing villous configuration lined by mucus-producing cells (Haematoxylin and eosin, ×40)

Microscopic examination revealed mucosa with villous and papillary infoldings, mostly covered by a single layer of tall columnar mucous-producing cells [Figures 1 and 2]. Stratification was seen in some areas. At places, mild dysplastic change was also seen in the basal half of the mucosa. Mucous secretion was present, but without distension of the lumen of appendix. The remaining layers showed presence of acute inflammation. These findings were mainly seen in the sections taken from the tip of the appendix. The base of the appendix (surgical margin) was normal. Based on the histopathological findings, a diagnosis of villous adenoma of appendix with mild dysplasia and acute appendicitis was made.

Appendiceal tumors account for less than 0.4% of neoplasms in the gastrointestinal tract and are found in less than 1% of appendectomies.[1,2] Adenoma of the appendix is a rare pathological entity, which may progress to invasive carcinoma. They rarely produce clinical symptoms, and are mostly discovered incidentally during histopathological examination. A few may present with acute appendicitis.[1,3]

Villous adenoma of the appendix is a rare pathological condition.[4,5] Adenomas of the appendixes, like adenomas elsewhere in the large intestine, are neoplastic lesions, which may progress to invasive adenocarcinoma,[3] and, once diagnosed, indicates a need for further investigation due to an association with neoplasia elsewhere.[6]

Appendiceal adenomas are diffuse villous lesions involving large areas of the appendiceal mucosa. They frequently have a villous configuration with papillary fronds covered by large number of mucus-producing cells, which often have a deceptively innocuous appearance. The dysplastic nature of the lesion is best appreciated by examining the epithelial characteristics in the basal half of the mucosa, where crowding and stratification of the cells with nuclear atypia will be appreciated. Areas with a serrated pattern may be seen. Excessive mucous production by the lining epithelial cells may occur, in which case a mucocoele of the appendix may form.[3]

Adenomas of the appendix may be cured by appendicectomy,[1,3] provided the resection line at the base of the appendix is tumor-free. Careful scrutiny of the remainder of the large intestine is necessary in patients with appendiceal adenomas because of the strong association with synchronous or metachronous colorectal adenomas and carcinomas.[3] Our patient has been asymptomatic for the last 2 years.
